# A natural mutation of the *NST1* gene arrests secondary cell wall biosynthesis in the seed coat of a hull-less pumpkin accession

**DOI:** 10.1093/hr/uhac136

**Published:** 2022-06-16

**Authors:** Xiaolong Lyu, Lu Shi, Meng Zhao, Zhangping Li, Nanqiao Liao, Yiqing Meng, Yuyuan Ma, Yulan Zhou, Qin Xue, Zhongyuan Hu, Jinghua Yang, Mingfang Zhang

**Affiliations:** Laboratory of Germplasm Innovation and Molecular Breeding, College of Agriculture and Biotechnology, Zhejiang University, Hangzhou 310058, China; Laboratory of Germplasm Innovation and Molecular Breeding, College of Agriculture and Biotechnology, Zhejiang University, Hangzhou 310058, China; Laboratory of Germplasm Innovation and Molecular Breeding, College of Agriculture and Biotechnology, Zhejiang University, Hangzhou 310058, China; Laboratory of Germplasm Innovation and Molecular Breeding, College of Agriculture and Biotechnology, Zhejiang University, Hangzhou 310058, China; Laboratory of Germplasm Innovation and Molecular Breeding, College of Agriculture and Biotechnology, Zhejiang University, Hangzhou 310058, China; Laboratory of Germplasm Innovation and Molecular Breeding, College of Agriculture and Biotechnology, Zhejiang University, Hangzhou 310058, China; Laboratory of Germplasm Innovation and Molecular Breeding, College of Agriculture and Biotechnology, Zhejiang University, Hangzhou 310058, China; Laboratory of Germplasm Innovation and Molecular Breeding, College of Agriculture and Biotechnology, Zhejiang University, Hangzhou 310058, China; Laboratory of Germplasm Innovation and Molecular Breeding, College of Agriculture and Biotechnology, Zhejiang University, Hangzhou 310058, China; Laboratory of Germplasm Innovation and Molecular Breeding, College of Agriculture and Biotechnology, Zhejiang University, Hangzhou 310058, China; Key Laboratory of Horticultural Plant Growth, Development and Quality Improvement, Ministry of Agriculture, Hangzhou 310058, China; Laboratory of Germplasm Innovation and Molecular Breeding, College of Agriculture and Biotechnology, Zhejiang University, Hangzhou 310058, China; Key Laboratory of Horticultural Plant Growth, Development and Quality Improvement, Ministry of Agriculture, Hangzhou 310058, China; Laboratory of Germplasm Innovation and Molecular Breeding, College of Agriculture and Biotechnology, Zhejiang University, Hangzhou 310058, China; Key Laboratory of Horticultural Plant Growth, Development and Quality Improvement, Ministry of Agriculture, Hangzhou 310058, China

## Abstract

Hull-less pumpkins (*Cucurbita pepo* L.) are naturally occurring novel variants known as oilseed or naked-seeded pumpkins, and are characterized by the absence of a normal lignified seed coat. Due to a specialized seed coat structure, these variants serve as a good model for studying seed coat formation and simplify the processing of pumpkin seeds. However, causal genes for this hull-less trait still remain unknown. Here, by bulked segregant analysis and fine mapping, we found that mutation of a single gene, *NAC SECONDARY WALL THICKENING PROMOTING FACTOR 1* (*NST1*), accounts for the hull-less trait. A 14-bp sequence insertion in the *CpNST1* gene causes premature termination of *CpNST1* translation, leading to lack of secondary cell wall (SCW) biosynthesis in hull-less seed coats. *In situ* hybridization analysis provided further evidence for the role of *CpNST1* in pumpkin seed coat SCW biosynthesis. Interestingly, through secondary cell wall compositional analysis, we found that the main SCW components differed among cell layers in the seed coat. RNA-seq analysis indicated an upstream role of CpNST1 in the SCW biosynthesis network. Collectively, our findings provide mechanistic insight into seed coat SCW biosynthesis, and a target gene for breeders to introduce this hull-less trait for commercial exploitation.

## Introduction

The evolution of sexual reproduction systems and the emergence of seeds underlies the evolutionary success of the flowering plants. Upon fertilization, seeds are developed and encased in a protective maternal integument referred to as the seed coat. Differentiation of the seed coat entails some dramatic cellular structural changes during the development of the seed, including the sequential deposition of a specialized cell wall. Some specialized cell types develop secondary cell walls (SCWs) to strengthen the mechanical support of the seed coat and often play important roles in seed protection, nourishment, dormancy, and dispersal, which facilitated the land colonization of flowering plants [1–3]. Seed coat structure and cell wall composition have large influences on seed physiology, and these traits exhibit different evolutionary adaptations among species [4]. SCWs play critical roles in the mechanical strength of the seed coat [4]. The composition of the seed coat SCW varies among angiosperm species, and cellulose, hemicellulose, and lignin are the three main components [5]. In addition, the units of which the lignin in the seed coat is composed also differ among species [6, 7]. The importance of seed coat lignin for seeds is illustrated by the *transparent testa 10* mutant in *Arabidopsis*, in which seed coat lignin deposition is disturbed, leading to a reduction of the germination rate [8]. In addition, CELLULOSE SYNTHASE3 (CESA3), CESA5, CESA9, and CESA10 were found to be critical for secondary cellulose synthesis in seed coat epidermal cells [9–12]. Both cellulose and lignin are the main components of SCWs. However, compared with SCW studies in plant vegetative tissues, research on the regulatory mechanisms of seed coat SCWs has received less attention, and to date their genetic architecture remains unknown. In the last 20 years, genetic and molecular analyses in *Arabidopsis* have significantly contributed to our understanding of many characteristics of the seed coat. MUM4 was demonstrated to be essential in producing normal levels of mucilage, a process that is directly regulated by GL2 [1, 13, 14]. In *Arabidopsis*, the cumulative level of mucilage is essential for seed coat SCW formation. Using the loss of function phenotypes, TTG1, TTG2, GL2, and MYB61 were demonstrated to be essential for mucilage synthesis [1, 13–17].

Although seed coat lignification is important for internal embryo protection, the hard seed coat tissues are not conducive to the further processing of seeds. The seed decoration process is often laborious and expensive, and seed coats are often treated as useless by-products, causing a waste disposal problem. Hence, in modern breeding programs hull-less is an important breeding trait in seed crops, such as barley [18], maize [19, 20], legumes [21], oil palm [22], and wheat [ 23]. This hull-less trait is usually controlled by single or several genes. By quantitative trait locus (QTL)-seq, a 250 kb QTL was reported to be associated with papery hull domestication in *Coix* [24]. Nine candidate genes were identified to be responsible for shelling percentage, the weight of kernels as a percentage of the weight of pods, an important economic trait in peanut production [25]. The mutation of *tga1* (*teosinte glume architecture 1*) in teosinte (the progenitor of maize) causes glume thinning, which leads to exposed and readily utilized kernels in maize [20].

Hull-less pumpkins (*Cucurbita pepo* L.), also known as oilseed pumpkins or naked-seeded pumpkins, are characterized by having a thin membranous seed coat instead of the normal lignified seed coat. The hull-less trait in pumpkin, in which the seed coat totally lacks lignification, is promising as an ideal model for studying seed coat developmental biology. In addition, due to the advantage of requiring no decortication process, the emergence of the hull-less trait facilitates the economical production of pumpkin seed oil, thereby turning a pumpkin into a specialized oil crop [26]. Thus, fine mapping of the causative genes for the hull-less trait will help us to dissect the developmental process of seed coat lignification, further enabling plant breeders to precisely introduce this novel trait into other pumpkin varieties or even other seed crops, thereby making the decortication process unnecessary for food or oil production.

**Figure 1:**
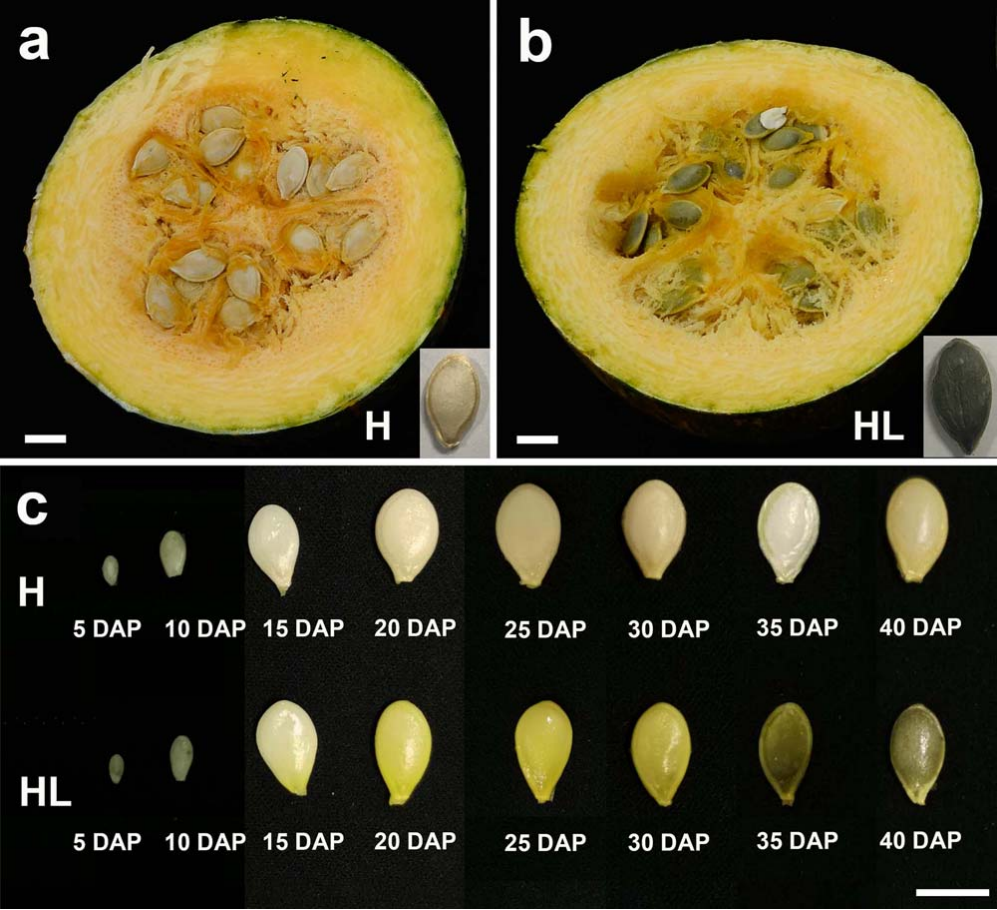
Phenotypic features of the seed coat in hulled and hull-less pumpkins. **a** Hulled pumpkins (H). **b** Hull-less pumpkins (HL). Hull-less seeds appear dark green as the outer seed coat layers have collapsed. **c** Characteristics of seed coat at different development stages in hulled and hull-less pumpkins. Observable phenotypes occurred at 20 DAP. Scale bar, 1 cm.

The seed coat of pumpkin normally consists of five zones with different cell types (epidermal, hypodermal, sclerenchyma, parenchyma, and chlorenchyma) [27, 28]. At maturity, the hypodermal, sclerenchyma, and partial parenchyma tissues will form an SCW providing mechanical support for the seed coat [27]. Hull-less pumpkins lack lignification in all seed coat layers and the outer four seed coat layers collapse at maturity. The collapse of these four seed tissue layers forms a paper-thin hyaline hull that reveals the green color of the innermost green chlorenchyma layer [28]. Hull-less pumpkins were first discovered in the southeast region of the Austro-Hungarian Monarchy in the 1880s [29]. Previous classic genetic studies on the hull-less seed phenotype indicated a major dominant gene controlling seed coat lignification [28]. However, these hull-less seeds show variation in the amounts of lignin deposited in the seed coat, varying from zero to intense lignification [29]. Earlier researchers made different assumptions concerning these variants, including regarding the trait as a consequence of a major recessive gene or a minor gene with modifiers, or postulating a polygenic model [26, 28–31]. Nevertheless, most researchers assume that a single gene mutation leads to the hull-less trait. Except for the apparently hull-less pumpkin, some normal seed lots exhibit different degrees of collapse in epidermal, hypodermal, and sclerenchyma tissues from the embedded mature seed coat. This is supposed to be a result of the seasonal variation in gene expression [27, 28]. Four enzymes, phenylalanine ammonia-lyase (PAL), cellulose synthase (CES), cinnamoyl CoA reductase (CCR), and 4-coumarate CoA ligase (4CL), may play important roles in testa lignification by regulating gene expression levels [32]. In 2012, Inan *et al*. [33] employed sequence-related amplified polymorphism (SRAP) and inter simple sequence repeats (ISSR) techniques in the molecular characterization of naked (hull-less seed) pumpkins [33]. However, to date, the causative gene(s) controlling the hull-less seed trait in pumpkins remain unknown. Recently, several reports of high-quality pumpkin genomes [34, 35] have facilitated the discovery of genes controlling important traits in pumpkins using a forward genetics approach. Genome-wide association studies (GWAS) and bulk segregant analysis (BSA) have been widely employed to dissect important traits in crops such as rice [36], tomato [37], cucumber [38], melon [39], and watermelon [40, 41].

In this study, by integrating BSA-seq and fine mapping tools, we uncovered the causal gene for the extant hull-less pumpkins in natural populations. The natural mutation of a single gene, *NAC SECONDARY WALL THICKENING PROMOTING FACTOR 1* (*NST1*), confers the novel hull-less trait. Our sequencing and fine-mapping results indicated that a 14-bp sequence insertion in the *CpNST1* gene caused premature termination of *CpNST1* translation, leading to a complete inability to biosynthesize SCWs in hull-less pumpkin seed coats.

## Results

### The hull-less trait in pumpkins is controlled by a single recessive allele

To investigate the inheritance pattern of the hull-less trait in pumpkins, we developed an *F*_2_ gene mapping population by crossing hulled (H) and hull-less (HL) pumpkins (Fig. 1). At maturity, the hull-less seeds appear dark green as the outer seed coat layers have collapsed, revealing the innermost green chlorenchyma layer (Fig. 1b).

The *F*_1_ hybrid seeds of H and HL pumpkins showed an apparently complete hulled phenotype, and nearly 3/4 (283/374) of the *F*_2_ plants bore hulled seeds, while 1/4 (91/374) were hull-less seeds (Table 1). The ratio of hulled seeds to hull-less seeds fitted a 3:1 Mendelian ratio (*χ*^2^ = 0.045, *P* = 0.832). Our genetic analysis showed that the hull-less trait was controlled by a single recessive allele (*hh*) in this population.

**Table 1:** Genetic analysis of hull-less phenotype in *F*_2_ population.

**Generation**	**Total number**	**Hulled**	**Hull-less**	**Expected ratio**	***χ*** ^**2**^ **-value**	***P*-value**
P-H	10	10	0			
P-HL	10	0	10			
*F*_1_	10	10	0			
*F*_2_ population	374	283	91	3:1	0.045	0.832

*χ* ^2^(0.05,1) = 3.841.

**Figure 2:**
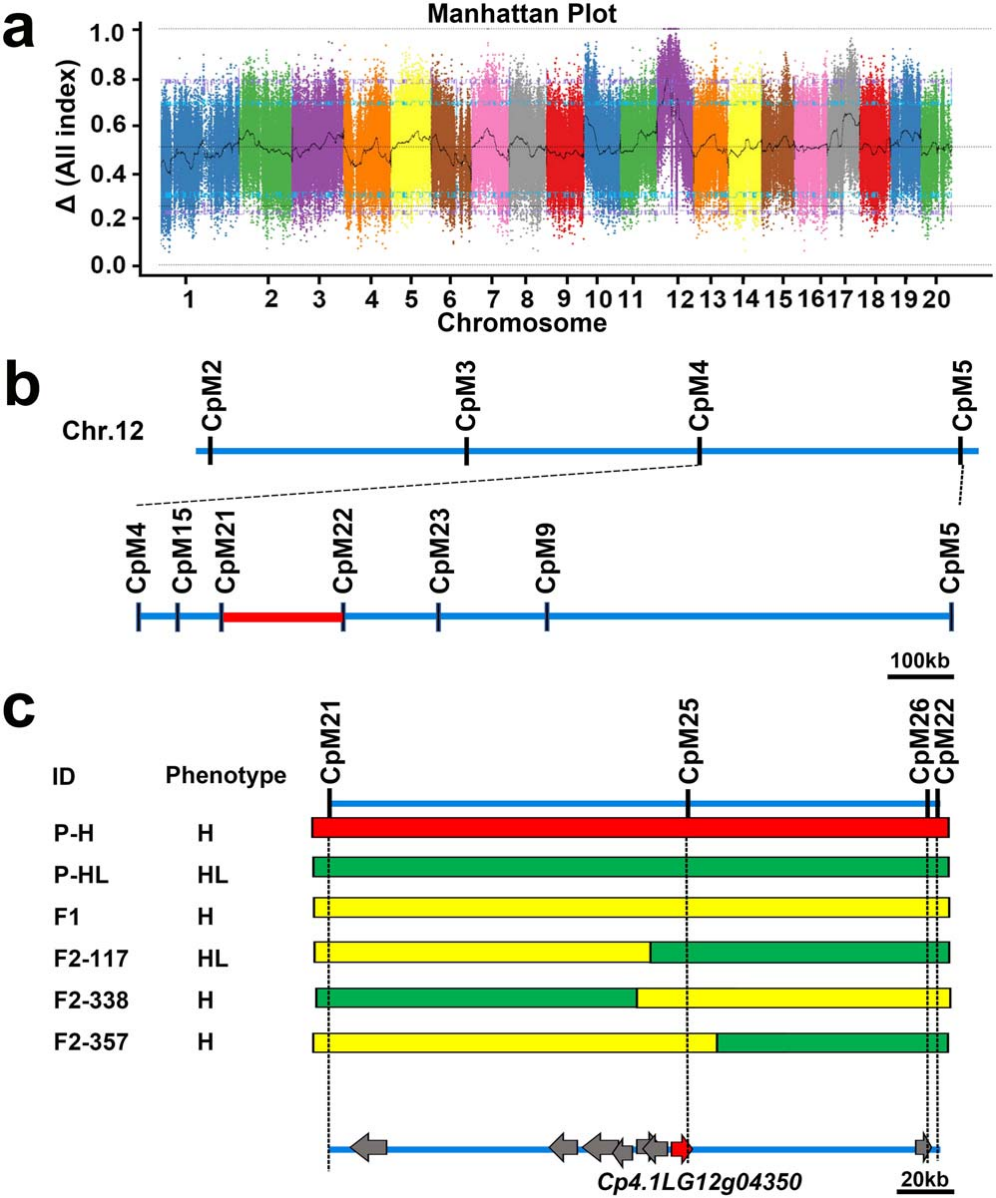
Fine mapping of the candidate gene for the hull-less trait. **a** Results of BSA of hull-less seed coat in pumpkin. Graph of the Δ All index values used for the hull-less trait association analysis. The *x*-axis indicates the 20 chromosomal positions, and the *y*-axis indicates the Δ All index. The blue line indicates the threshold line at 95% confidence interval and the purple line indicates at 99%. **b** High-resolution genetic mapping was performed by analyzing a chromosome segment substitution population of 367 *F*_2_ individuals segregating with the target region. The red box indicates the candidate region (189.67 kb) between molecular markers CpM21 and CpM22. Scale bar: 100 kb. **c** Further fine mapping of recombinant individuals using markers CpM25 and CpM26 located the candidate gene to *Cp4.1LG12 g04350*. Marker CpM25 is located in the indel variation region of *Cp4.1LG12 g04350*. Marker CpM26 is located in the non-synonymous SNP position of *Cp4.1LG12 g04330*. The seed coat phenotype of the recombinant individual *F*_2_-117 was the same as that of the parent HL (P-HL), having an identical hull-less seed coat phenotype. The seed coats of *F*_2_-338 and *F*_2_-357 recombinant individuals possess a lignified seed coat the same as that of parent H (P-H) and hybrid *F*_1_. Chromosomal segments are represented by solid bars filled in different colors. Red indicates the homozygous P-H (*HH*), green the homozygous P-HL (*hh*), and yellow the heterozygous (*Hh*).

### Fine mapping of the candidate locus for the hull-less trait

To anchor the candidate locus associated with the hull-less pumpkin phenotype, we employed BSA. Genomic DNA of 40 hulled pumpkins and 40 hull-less pumpkins (all *F*_2_ individuals) were mixed equally and set as the H pool and HL pool, respectively. DNA of the two parent pools (P-H and P-HL) and the two *F*_2_ mixed pools were sequenced, producing 27.078 Gb of clean data with Q20 ≥ 97.04%, Q30 ≥ 92.44%, and a 38.55–38.95% G/C ratio (Supplementary Data Table S1). Ultimately, we obtained approximately 2.87, 9.39, 9.80, and 11.02 Gb of clean reads from P-H, P-HL, the H-pool, and the HL-pool (Supplementary Data Table S1), respectively. The mapping rates were 98.00–98.24%, with 11.69×, 13.78×, 39.83×, and 44.20× average depths for P-H, P-HL, H-pool, and HL-pool. In total, we obtained 634 457 homozygous SNPs and indels between the P-H and P-HL pools. To identify SNPs/indels associated with the hull-less trait, the Δ All index was calculated by subtracting the SNPs/indels index values of the two *F*_2_-generation pools (Fig. 2a). Then, we identified peak regions above the threshold values (95 and 99%) as the candidate regions for the target hull-less trait according to the null hypothesis (Fig. 2a). To obtain more accurate candidate regions, we also performed a G′ value analysis of the two *F*_2_ pools (Supplementary Data Fig. S1). The two mapping results shared corresponding 5.04 Mb (Cp4.1LG Chr12: 400 001–5 686 000; 95% significance level) and 1.43 Mb (Cp4.1LG Chr12: 2 638 001–4 133 000; 99% significance level) overlapping regions in which the candidate gene could be located.

**Figure 3:**
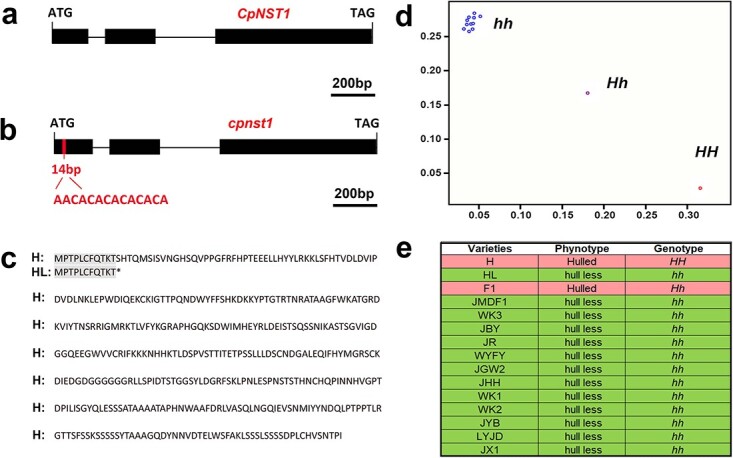
Gene structure, sequence translation, and genotyping variations in the *CpNST1* candidate gene between hulled and hull-less pumpkins (**a**–**d**). **a**, **b** Gene structure of *CpNST1* in hulled pumpkins (**a**) and the mutant allele *cpnst1* in hull-less pumpkins (**b**). Black boxes represent exons; intervals between exons are introns, and the 14-bp indel position is marked by a short vertical red line. Boxes and lines are scaled as indicated. **c** Amino acid sequences of CpNST1. A 14-bp indel insertion led to premature termination of translation in hull-less pumpkins. **d** The genotyping of NST1 in 10 hull-less pumpkins by KASP. The 14-bp indel KASP marker, CpM25, was used for genotyping. e The information of 10 hull-less pumpkin accessions.

To further fine map the possible candidate locus for the hull-less trait, we tailored Kompetitive allele-specific PCR (KASP) SNP markers in the candidate region (Supplementary Data Table S2) and constructed an SNP-based genetic map of the candidate region using the *F*_2_ population to narrow the location (Fig. 2a). Finally, using 24 KASP markers, we narrowed the target region to a 189.67-kb window (Cp4.1LG Chr12: 3 287 529–3 481 749) by three critical recombinants from 367 *F*_2_ individuals (Fig. 2b). This region hosts eight genes, but only two genes (*Cp4.1LG12 g04330* and *Cp4.1LG12g04350*) contained a non-synonymous SNP or indel mutation in the exons. Then, we designed another two molecular markers, CpM25 located in the exonic indel variation region of *Cp4.1LG12g04350* and CpM26 located in the non-synonymous SNP position of *Cp4.1LG12g04330*, for further fine-mapping using recombinant individuals. Finally, we obtained *Cp4.1LG12 g04350* as the candidate (Fig. 2c).

### A 14-bp insertion mutation of CpNST1 confers the novel hull-less trait

*Cp4.1LG12g04350* was predicted to encode an NAC transcription factor. According to the BLASTp and phylogenetic tree analysis, *Cp4.1LG12g04350* is a homolog of *Arabidopsis NAC SECONDARY WALL THICKENING PROMOTING FACTOR 1* (*NST1*; Supplementary Data Fig. S2S2). Sequence analysis showed that, compared with the hulled pumpkin allele of *CpNST1*, there is a 14-bp indel (AACACACACACACA) insertion after T in the hull-less pumpkin (Fig. 3a–c, Supplementary Data Figs S3 and S4). This indel insertion led to the premature emergence of the stop codon TAA (Fig. 3a–c, Supplementary Data Figs S3 and S4), which terminated protein translation and caused a probable function loss. To further test this hypothesis, the 14-bp indel KASP marker CpM25 was used for genotyping the candidate *CpNST1* in the 367 *F*_2_ individuals and 10 hull-less germplasm accessions. The resulting genotyping results were completely in accordance with the observed phenotype (Supplementary Data Table S3, Fig. 3d and e), supporting the hypothesis that the 14-bp insertion in the first exon of *CpNST1* caused the hull-less trait mutation.

### Failure of the secondary cell wall formation in the hull-less seed coat

**Figure 4:**
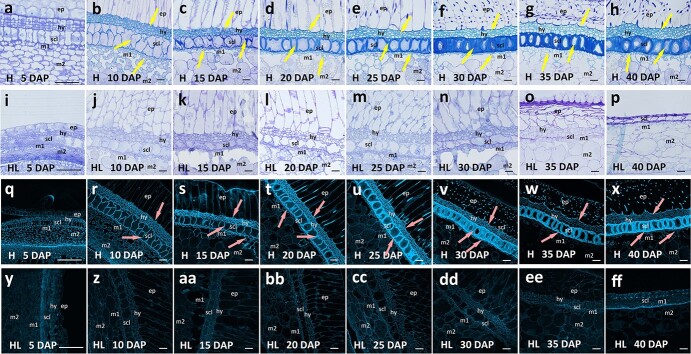
Microstructural changes in the developing seed coat in hulled (**a**–**h, q**–**x**) and hull-less (**i**–**p, q**–**ff**) pumpkins at various days after pollination (DAP). SCWs were deposited in the pitted subepidermal (hy), scl (sclerenchyma), and m1 (pitted parenchyma) cell layers in hulled pumpkins (**a**–**h, q**–**x**), starting from 10 DAP (**b** and **r**), while no SCWs formed in hull-less pumpkin seed coats during the entire seed developmental process (**i**–**p, q**–**ff**). Arrows indicate cells with SCWs. ep, ribbed palisade cells of the epidermis; m2, reticulated spongy parenchyma. Scale bars, 25 μm.

**Figure 5:**
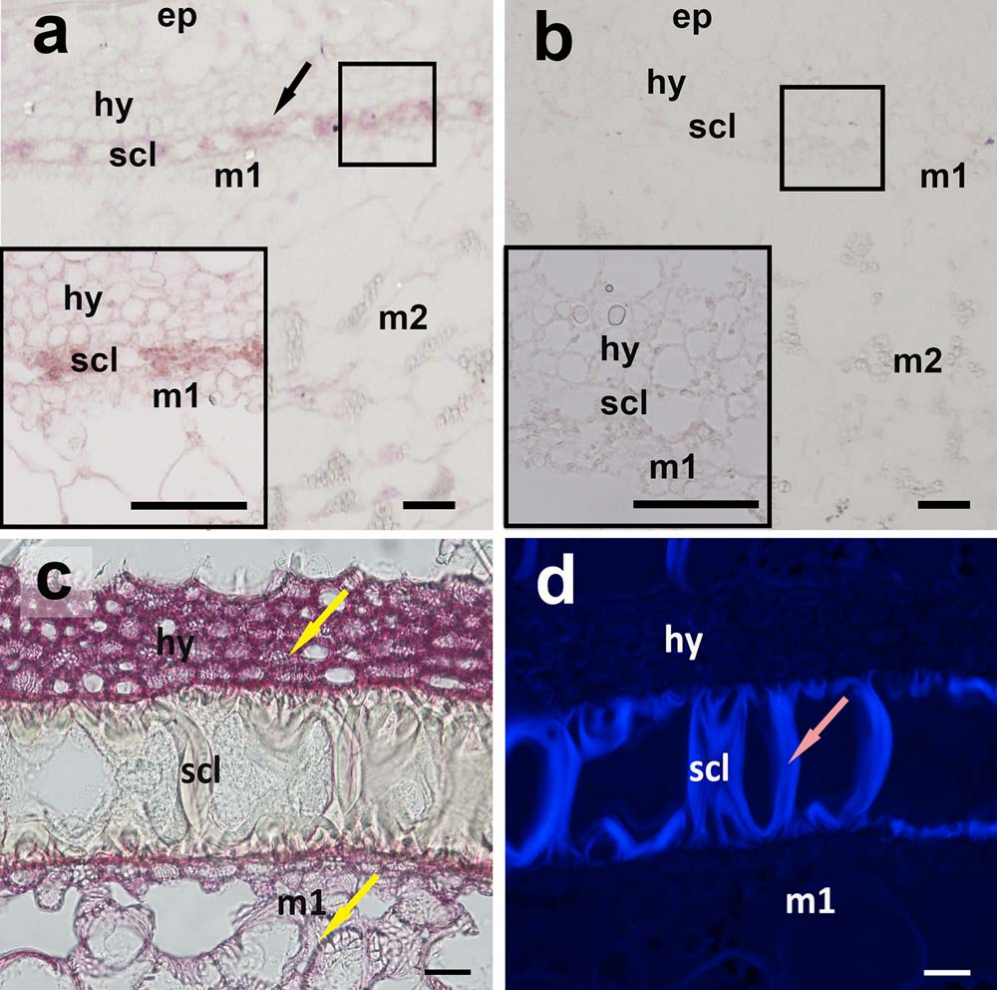
CpNST1 *in situ* detection and seed coat SCW composition analysis. **a**, **b** CpNST1 *in situ* expression at 15 DAP in the hulled seed coat. The arrow indicates the specific expression of *CpNST1* in the pitted subepidermal (hy), sclerenchyma (*scl*), and pitted parenchyma (m1) cell layers, especially in scl (**a**). The negative control is shown in (**b**); zoom-in views of the three cell layers are shown in the boxes at bottom left. **c** Lignin was specifically stained with phloroglucinol and presented a red color under white light. The yellow arrows indicate the cell wall deposited lignin. **d** Cellulose was stained with calcofluor white and presented a blue color under blue laser light (300–440 nm). Staining results indicated that hy and m1 were composed of lignin, while scl was primarily composed of cellulose. The pink arrow indicates the cellulose-rich cell wall. m2, reticulated spongy parenchyma. Scale bars, 25 μm.

In the model plant *Arabidopsis*, NST1 is known to play key roles in regulating secondary cell wall biosynthesis [33]. To characterize the hull-less trait controlled by the recessive mutation of *CpNST1*, we conducted a sequential anatomical analysis of the seed coat at different developmental stages in hulled and hull-less pumpkins. Compared with the hulled seed coats, no loss of cell layers was observed, and all cells developed to the normal size in hull-less seed coats (Fig. 4). As expected, the only difference was the lack of SCW formation in cell types of the hull-less seed coat (Fig. 4i–p and y–ff). In contrast, in hulled pumpkins, three cell layers, the pitted subepidermal (hy), sclerenchyma (scl), and pitted parenchyma (m1) layers, initiated the formation of SCWs 10 days after pollination (DAP) (Fig. 4a–h and q–x). To confirm the involvement of *CpNST1* in the SCW thickening process of the seed coat, we designed a specific RNA probe of *CpNST1* for *in situ* hybridization analysis in hulled seed coats. The hybridization results demonstrated that *CpNST1* was primarily expressed in the hy, scl, and m1 cell layers, and especially in the scl layer (Fig. 5a and b). These results supported the hypothesis that CpNST1-mediated SCW formation is required for the development of the seed coat in pumpkin seeds.

In plants, SCWs are usually composed of cellulose, hemicelluloses, and lignin [34]. To characterize the SCW composition of the three different cell layers of the pumpkin seed coat, the hulled seed coat sections were stained with phloroglucinol to detect lignin or calcofluor white to detect cellulose. Surprisingly, we found that the SCWs of the three cell layers were composed of different components. The cell wall of hy and m1 layers was composed of deposited lignin (Fig. 5c), while the scl cell layer was mainly composed of cellulose (Fig. 5d), indicating that CpNST1 might regulate SCW biosynthesis in different cell layers through different pathways.

### Transcriptomic profiles associated with SCW regulatory network in hull-less seed coat

**Fig. 6:**
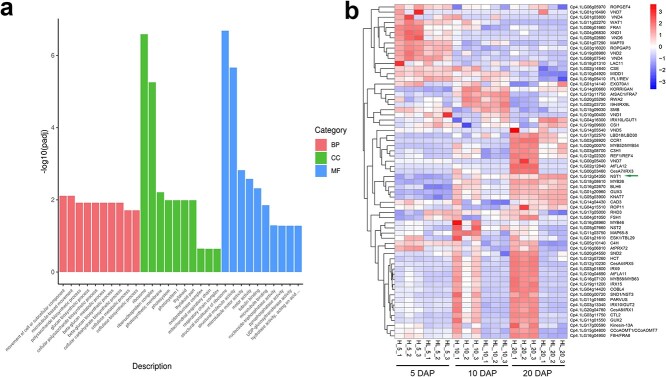
RNA-seq analysis during seed coat development in hulled and hull-less pumpkins. **a** GO annotation and enrichment of DEGs between hulled and hull-less pumpkin seed coats at 20 DAP. BP, biological process; CC, cellular component; MF, molecular function. **b** Gene expression patterns of orthologous genes involved in SCW biosynthesis in hulled and hull-less pumpkin seed coats at 5, 10, and 20 DAP. Compared with hull-less pumpkins, many orthologs were upregulated at 10 DAP in hulled pumpkin seed coats. Three biological replicates were used to analyze the data.

In *Arabidopsis*, the biosynthesis network of SCWs exhibits a series of regulatory cascades, and NAC TFs usually act as key upstream regulators [42]. In this study, according to the analysis of seed coat cell wall components, loss of function of CpNST1 led to the inability to deposit both lignin and cellulose. To identify the genes participating in the CpNST1-mediated seed coat SCW biosynthesis network, we performed a transcriptome analysis during seed coat development. According to the SCW biosynthesis timeline (Fig. 4), we collected 5, 10, and 20 DAP seed coat samples of both hulled and hull-less pumpkins to perform an RNA-seq analysis that produced 121.96 Gb of clean data with Q20 ≥ 98.00%, Q30 ≥ 93.13%, and a 45.07%–45.6% G/C ratio (Supplementary Data Table S3). Then, we performed differential gene expression (DEG) analysis between hulled and hull-less pumpkins at different developmental stages. As shown in Supplementary Data Fig. S5, the number of differentially expressed genes increased with the development of the seed coat, consistent with the phenotypic differences of seed coat development (Fig. 4). We then performed Gene Ontology (GO) enrichment of the DEGs between hulled and hull-less seed coats. At 20 DAP we found the GO terms of the biological process categories ‘cellulose biosynthesis process’, ‘cellulose metabolic process’, ‘glucan biosynthetic process’, ‘beta glucan biosynthetic process’, ‘beta glucan metabolic process’, ‘polysaccharide biosynthetic process’, and ‘cellular polysaccharide biosynthetic process’ were most significantly enriched (Fig. 6a), indicating that the cellulose biosynthesis pathway was interrupted in the hull-less seed coat phenotype.

In *Arabidopsis*, many TFs and enzymes have been reported as being involved in the SCW biosynthesis network in different tissues, including anthers, siliques, and stems [43]. To determine whether these genes were also involved in SCW development in the seed coat, we analyzed the orthologous genes in *C. pepo* that have been demonstrated to be involved in SCW biosynthesis in *Arabidopsis* (Supplementary Data Table S4, Fig. 6b) and determined the gene expression patterns during seed coat development. As expected, in hulled pumpkins the expression of many genes gradually increased to high levels at 20 DAP (Fig. 6b), which was consistent with the deposition timing of the secondary cell walls (Fig. 4a–h and q–x), while in hull-less seed coats these genes remained at very low expression levels during seed coat development (Fig. 6b). Among the DEGs, all secondary cellulose biosynthesis or assembly genes showed significantly lower expression levels in hull-less seed coats; these included *CesA4*, *CesA7*, and *CesA8*, which have been reported as playing downstream roles in NST1 [44]. Five lignin biosynthesis and polymerization genes, *HCT*, *C4H*, *FSH1*, *CCoAomt7*, and *PRX72*, presented high expression patterns during seed coat development in hulled pumpkins, indicating that they may participate in lignin deposition in the hy and m1 layer cells of the pumpkin seed coat. In addition, two NST1-downstream TFs, MYB58 and MYB46 [45, 46], also showed a DEG pattern, suggesting that they may participate in seed coat SCW biosynthesis in pumpkins. Interestingly, according to the gene expression analysis, all three NST TFs were involved in seed coat SCW biosynthesis pathways, but the expression levels of both *NST2* and *NST3* were affected if *NST1* displayed loss of function (Supplementary Data Fig. S2, Fig.S26b). These results suggest that CpNST1 plays a key role in the pumpkin seed coat SCW regulatory network, and its loss of function will interrupt this regulatory network (Fig. 6).

## Discussion

Seeds contributed to the successful land colonization of flowering plants. Such colonization advantages were to a large extent achieved by the emergence of a seed coat that protects and facilitates the dispersal of the zygotic embryo [3]. The mechanical strength of the seed coat is primarily achieved by the deposition of SCWs in sclerenchyma cells. SCWs are composed of cellulose, hemicelluloses (xylan and glucomannan), and lignin [42]. In *Arabidopsis*, most studies of SCWs in the seed coat have focused on cellulose biosynthesis. CELLULOSE SYNTHASE9 (CESA9) has been demonstrated to be involved in SCW synthesis in *Arabidopsis* epidermal testa cells [9]. CESA3, CESA5, and CESA10 are essential for cellulose biosynthesis in seed coat epidermal cells, and they also affect the extrusion of mucilage [10–12]. To date, owing to the unavailability of seed coat mutants, systematic studies on the SCWs of the seed coat are scarce. Several mucilage synthesis transcription factors, including MUM, TTG1, GL2, MYB61, and TTG2, have been reported to participate in seed coat SCW biosynthesis [1, 13–17]. Here, anatomical analysis of the seed coat supported the suggestion that hull-less pumpkins are an ideal experimental model for dissecting seed coat SCW formation; in this model plant system no SCWs develop in any of the relevant cell types, but all cells can develop to normal size (Fig. 4). The single phenotypic difference in hulls facilitates the fine-mapping of the candidate genes/QTLs that control the biosynthesis of seed coat SCWs.

Although the seed coat is important for plant seed protection, seed coats as by-products are less effectively and economically utilized in seed crops, since the decortication processes are laborious and expensive, and require waste disposal. Hence, hull-less and even hull-free cultivars are highly preferred in the breeding of many crops, including barley [18], maize [19, 20], legumes [21], oil palm [22], and wheat [23]. The hull-less or naked seed trait is usually controlled by a single gene or several genes in many seed crops [18–23]. Undoubtedly, the discoveries of these loci will make important contributions to seed crop breeding. Pumpkins are commonly consumed as vegetables. The emergence of a mutant (hull-less pumpkin) exhibiting a naked seed trait (no seed coat) turns the pumpkin into an important oil crop without the need for decortication [29, 47]. The recent publication of a high-quality pumpkin genome [34–35] has enabled the discovery of genes controlling important traits in pumpkins. Combining QTL-seq and fine mapping toolkits has been shown to be an effective and efficient way to identify target QTLs/genes for important crop traits [41]. In this study, we employed BSA-seq and haplotype analysis and successfully anchored a single gene, *CpNST1*, that is responsible for the hull-less trait (Fig. 3). This mutation-induced hull-less seed is an important trait, providing a way to skip the decortication process for the commercial use of pumpkin seed. The hull-less seed trait is exploited for oil extraction and for snacks due to its high nutritional and medicinal value [30]. Identification of the causative gene for the hull-less trait provides a target gene for breeders to introduce this valuable hull-less trait into pumpkins for commercial breeding.

In the model plant *Arabidopsis*, *NST1* is known to play key roles in anther dehiscence and pod shattering via regulating SCW synthesis [48, 49]. The homologs of *NST1* in the moss *Physcomitrella patens* were shown to be essential in the early formation of water-conducting systems during plant evolution [50]. NST1 as an upstream TF that usually participates in SCW biosynthesis by regulating other TFs [43]; for example, KNAT7, MYB46, MYB58, MYB63, MYB83, MYB103, and SND3 have been demonstrated to be direct targets of NST1 [46, 51–53]. Here, via fine-mapping, as well as anatomical and *in situ* hybridization analyses, we revealed a new role of NST1 in plant SCW biosynthesis that has never been reported to be involved in plant seed coat development. The divergent role of NST1 in pumpkin plants could be related to species differences.

The regulatory network for the plant SCW synthesis is complex and involves many transcription factors and enzymes [5, 43]. The differential composition of SCWs was observed in different cell layers of the pumpkin seed coat (Fig. 5b and c), suggesting that CpNST1 may participate in regulating SCW biosynthesis in the pumpkin seed coat through multiple pathways. Interestingly, the RNA-seq analysis identified many reported cellulose biosynthesis and assembly genes as well as lignin biosynthesis and polymerization genes (Fig. 6b, Supplementary Data Table S4) that may participate in the pumpkin seed coat SCW biosynthesis network. These genes may play roles downstream of CpNST1. Among these genes, TFs and enzymes such as *MYB46*, *MYB58*, *CesA4*, *CesA7*, and *CesA8* have been shown to be directly or indirectly regulated by NST1, participating in SCW biosynthesis in different *Arabidopsis* tissues besides the seed coat [44, 46]. The current finding serves as a good exemplar for the genetic toolkit study of conserved homologous genes that regulate similar but different morphological phenotypes consistently across different species. In addition, through the expression analysis of pumpkin-homologous genes related to SCW biosynthesis in the *Arabidopsis* in the seed coat, we found several TFs and enzymes participating in pumpkin seed coat SCW biosynthesis (Fig. 6b). Together with cell wall composition analysis of the different cell layers (Fig. 5b and c), we concluded that there is more than one pathway regulating pumpkin seed coat SCW biosynthesis. Loss of function of any gene in these pathways could lead to defects in the development of SCWs in different cell layers of the seed coat. In addition, from RNA-seq analysis, we found the expression level of *CpNST1* at 20 DAP in the HL even higher than that in the H cultivar. This phenomenon was also found in previous research about a loss-of-function point mutation in the *NST1* gene of *Medicago truncatula* that leads to increased *NST1* expression [54]. Scientists assume that there is an unknown upstream signal that turns on the *NST1* promoter in the absence of functional NST1 protein [54]. In addition, the expression of the NAC master switch itself is under both positive (autoregulatory) and negative control [54]. Here, we suppose that there might be a negative feedback loop in the pumpkin seed coat SCW biosynthesis pathway. The absence of non-functional CpNST1 in HL stimulated its high expression. Collectively, the discovery of potential CpNST1 downstream genes provides important clues for characterizing the mechanism of pumpkin seed coat SCW formation. We believe that the discovery of a new function of CpNST1 in pumpkin seed coat formation will shed light on hull-less pumpkins as a model for studying SCW formation across species.

## Materials and methods

### Plant materials

Our laboratory-preserved *C. pepo* accessions H (hulled pumpkin) and HL (hull-less pumpkin) were selected as parent lines. The *F*_2_ population was obtained by hybridization of H and HL and subsequent self-crossing. Parents and 400 *F*_2_ seedlings were raised in a greenhouse in Hangzhou City, China, in the spring of 2020. Mature seeds were harvested at 40 DAP for seed coat trait investigation. Ten hull-less germplasm accessions from different sib families were grown in 3:1 mixed peat:turface medium in a greenhouse on the Zijingang campus.

### DNA extraction and library construction

Total genomic DNA was extracted from fresh leaves of young seedlings using the CTAB method (VWI, China) as described in Liao *et al*. [41]. The *F*_2_ offspring H-pool (hulled pumpkins) and HL-pool (hull-less pumpkins) were constructed by mixing 40 hulled and 40 hull-less *F*_2_ individuals. A TruSeq Nano DNA LT Sample Prep Kit (Illumina, USA) was used to construct sequencing libraries. All libraries were sequenced on an Illumina HiSeq 4000 platform. The sequencing data quality was determined by FASTQC [55]. The QC standard pipelines were employed as described in Liao *et al* [41].

### Bulked segregant analysis pipelines

The *C. pepo* (zucchini) genome (http://cucurbitgenomics.org/organism/14) [35] was used as a reference genome for sequence alignment. SAMtools software was used to convert BAM files. SNP calling and indel filtering were performed using the Unified Genotyper and the Variant Filtration (−cluster Window Size 4，−filter Expression ‘QD < 4.0 || FS > 60.0 || MQ < 40.0’, −G_filter ‘GQ < 5’) by GATK3.8 software [56]. Then, the annotation of SNPs and indels was performed using ANNOVAR (version 20 200 316) software based on the GFF3 files of the reference genome. All homozygous SNPs and indels between two parents were extracted from the VCF files. The read depth information for homozygous SNPs/indels in the offspring pools was obtained to calculate the SNP/indel index [57]. The genotype of one parent was used as the reference and the statistic read number of this reference parent in the offspring pool was calculated. Then the ratio of different reads to the total number was calculated, which represented the SNP/indel indexes of the base sites. The SNP/indel indexes in both pools were filtered to <0.3 and the sites with SNP depth <7 were filtered out. The SNP/indel index of the whole genome was calculated using sliding window methods, with a window size of 1 Mb and a step size of 10 kb as the default settings. The difference in the SNP/indel indexes between two pools was calculated as the Δ(SNP/indel) index. The G′ value was calculated using the QTL-seq package [41].

### An SNP-based target region genetic map using KASP

To narrow the target region for fine mapping, we constructed a genetic map for the target region using 367 *F*_2_ individuals via the KASP platform. The primer combinations (Fam, Hex, R) were used as markers for genotyping and genetic map construction (Supplementary Data Table S2). The KASP reaction was performed as described in Liao *et al.* [41]. After amplification, an LGC Genomics system (Hoddesdon, UK) was used to derive the genotyping results.

### Bright-field and fluorescence microscopic observation

Hulled and hull-less pumpkin seed coats were collected, fixed, embedded, and sliced as described in the protocol [58]; 2 μm-thick sliced sections were prepared and stained with 0.1% Toluidine Blue O at room temperature for 1 minute, then washed with clean ddH_2_O and covered with a cover glass. Images were taken with a Nikon Fluorescence Scanning Confocal Microscope (Nikon, Japan) and a Nikon DS-Ri1 microscope (Nikon, Japan).

### *In situ* hybridization histochemistry assay

Hulled pumpkin seed coats were collected, fixed, and embedded following protocols in Nikovics *et al.* [59]. Ten micrometer-thick sliced sections were prepared. The *in situ* hybridization was performed as in Nikovics *et al.* [59] with minor modification. Briefly, sections were deparaffinized in 100% Histo-clear (USA), followed by dehydrating in an ethanol gradient of 100, 95, 85, 70, 50, 30, and 0% for 5 minutes each. Then, proteinase K (μg/mL) working solution was added for incubation at 37°C for 5 minutes. This was followed by adding pre-hybridization solution and incubating for 1 hour at 37°C and then incubating with 1 μM digoxin-labeled riboprobe (*CpNST1*: GGCTGTCCAGGGTTTTGTGGTTTTTCTT) hybridization solution at 42°C overnight. The sections were then washed with a gradient of 2×, 1× and 0.5× SSC for 5 minutes each at 37°C. Then, after incubating in the blocking solution (rabbit serum) at room temperature for 30 minutes, the sections were incubated with anti-DIG-HRP at 37°C for 40 min. Freshly prepared NBT/BCIP chromogenic reagent was used to mark the tissue. The sections were natural air-dried and mounted with glycerol jelly mounting medium. Images were taken with a Nikon DS-Ri1 microscope (Nikon, Japan).

### Differential secondary cell wall component staining

Hulled pumpkin seed coats were collected at 25 DAP and then fixed and embedded in paraffin. Ten micrometer-thick sliced sections were prepared for subsequent staining. The sections were stained with phloroglucinol to detect lignin, or calcofluor (with 10% potassium hydroxide) white stain to detect cellulose.

### RNA-seq analysis

Total RNA was extracted from seed coats of hulled and hull-less pumpkins at 5, 10, and 20 DAP using a Trizol kit (Invitrogen, USA). Then, 1 μg of RNA from each sample was taken for sequencing using an Illumina HiSeq 2000 platform. Three biological replicates were performed for each stage. Transcriptome analysis was performed as described by Trapnell *et al.* [60]. The clean reads filtered from raw data were mapped onto the *C. pepo* (zucchini) reference genome (http://cucurbitgenomics.org/organism/14) [26]. Low-quality reads (unknown nucleotides >5% or low Q value ≤20%) were removed. FPKM (fragments per kilobase of transcript per million mapped reads) values were calculated to estimate gene expression levels by Cufflinks software [60]. DEGs were determined using the edgeR package of R software [61], and the false discovery rate ≤.05 and the absolute value of log_2_ fold change ≥1 (|log_2_ FC| ≥ 1) were used as the threshold to determine statistically significant differences in gene expression [62].

### Expression of secondary cell wall biosynthesis-related genes

To analyze the expression levels of putative genes involved in SCW biosynthesis, protein sequences of genes involved in *Arabidopsis* SCW biosynthesis [34, 51] from the TAIR database (https://www.arabidopsis.org/) were used to identify candidate orthologs in *C. pepo*. The key criteria of BLASTP were an E-value <1e−5 and identity >50%. Then, the protein domains of the candidate genes were identified using HMMER software, and the genes with different domains were filtered. The expression levels of the candidate orthologs (Supplementary Data Table S5) were obtained from the RNA-seq data. A heat map of these genes was generated using an R script based on normalized read FPKM values of all genes transformed to log_2_ (value +1).

### Phylogenetic analysis

*Arabidopsis* NAC transcription factor protein sequences were downloaded from the TAIR database and were used to search for pumpkin (*C. pepo*) homologs using BLASTP (E-value <1e−5 and identity >50%). All the putative candidates were manually verified by HMMER software to confirm the presence of the NAM conserved domain. Multiple sequence alignments of the 227 NAC TF amino acid sequences were performed, and the unrooted phylogenetic trees were constructed according to the maximum likelihood method using FastTree Version 2.1.10.

## Acknowledgements

This work was supported by the National Key Research and Development Plan of China (2019YFD1001904) and the Key Science and Technology Program for Agricultural (Vegetable) New Variety Breeding of Zhejiang Province (2021C02065). We thank Novogene for providing technical support for sequencing. We also thank LetPub (www.letpub.com) for its linguistic assistance during the preparation of this manuscript.

## Author contributions

M.Z. and X.L. conceived and designed the experiments. X.L., L.S., M.Z., Z.L., and N.L. conducted most of the experiments; Y. Meng, Y. Ma, Y.Z., Q.X., Z.H. and J.Y. performed some of the field work and analysis; X.L. wrote the manuscript and M.Z. revised it.

## Data availability

The RNA-seq data have been deposited in Gene Expression Omnibus (GEO) under accession number GSE205063. Other data generated in this study are included in this article and its supplementary information files.

## Conflict of interest

The authors declare that they have no conflict of interest.

## Supplementary data

Supplementary data is available at *Horticulture Research* online.

## Supplementary Material

supp_data_uhac136
