# Supplementary figures and images for: A natural mutation of the NST1 gene arrests secondary cell wall biosynthesis in the seed coat of a hull-less pumpkin accession
Source: Hortic Res. 2022 Jun 16;9:uhac136. doi: 10.1093/hr/uhac136 (PMC9437724; doi:10.1093/hr/uhac136)

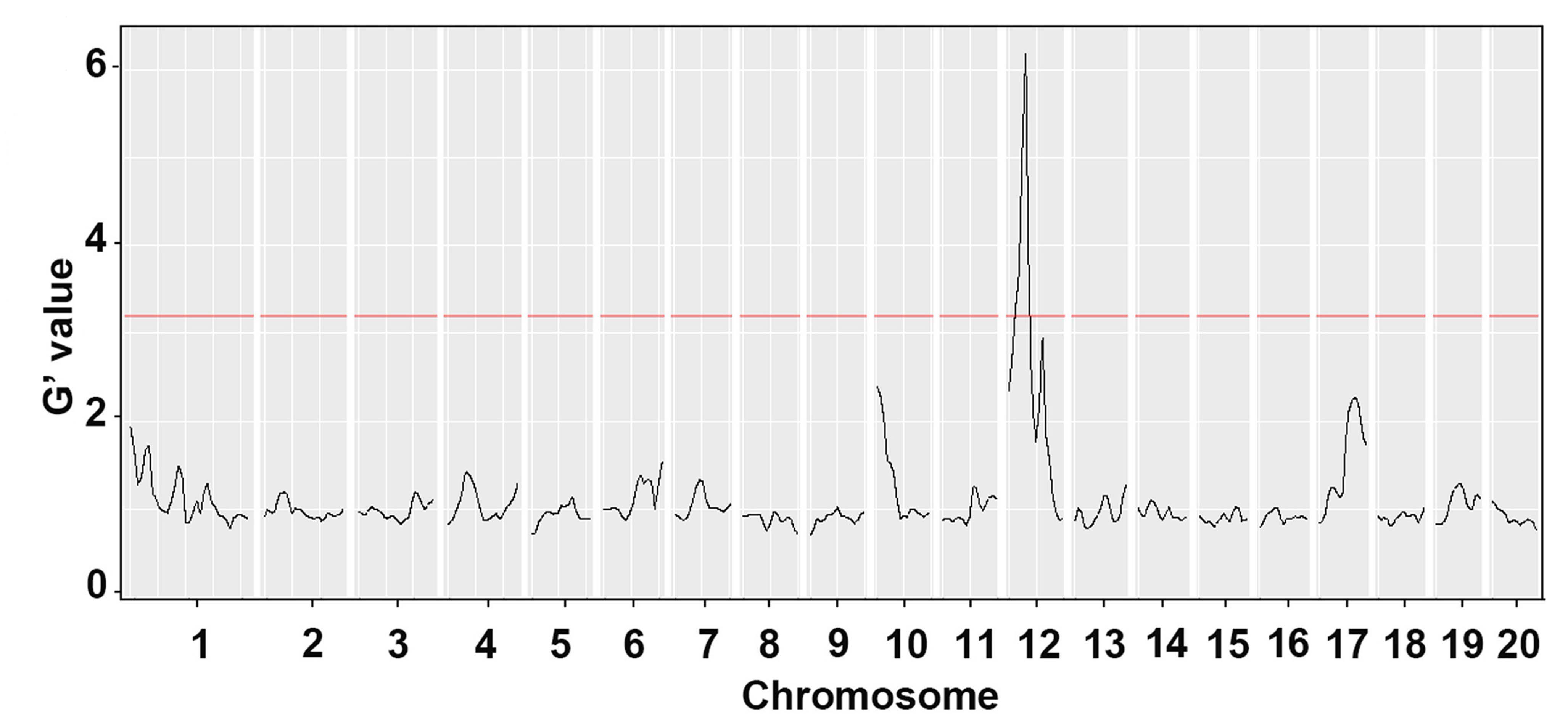

Supplement: supp_data_uhac136 [file supp_data_uhac136.zip › Fig. S1.tif]

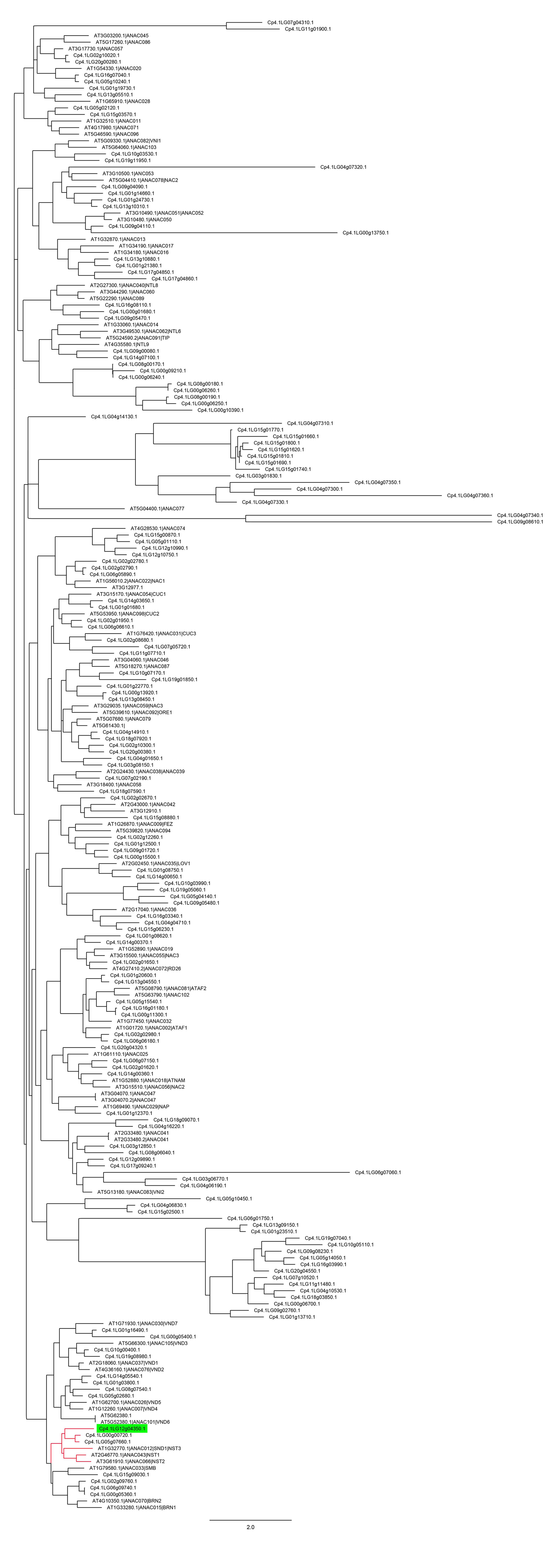

Supplement: supp_data_uhac136 [file supp_data_uhac136.zip › Fig. S2.tif]

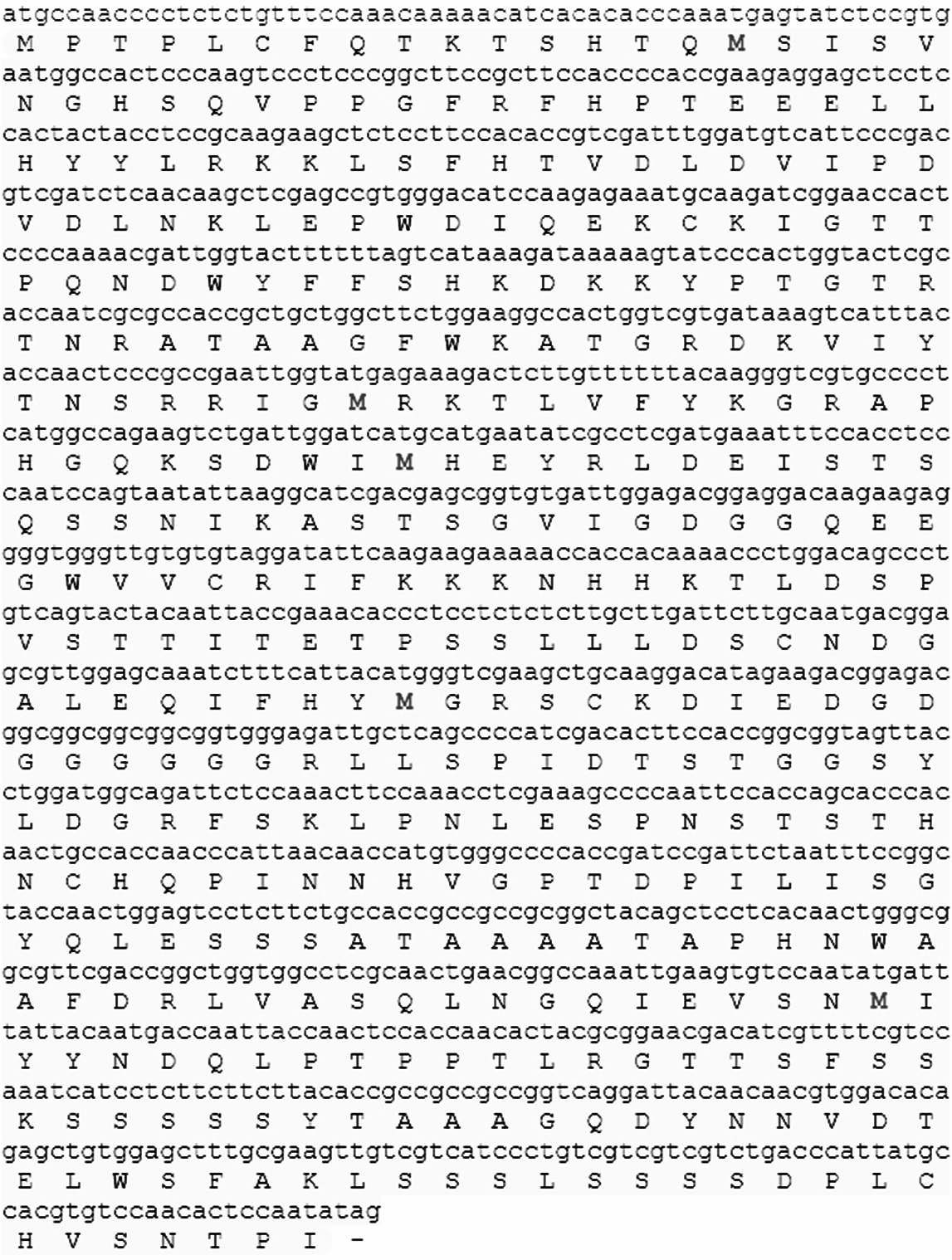

Supplement: supp_data_uhac136 [file supp_data_uhac136.zip › Fig. S3.tif]

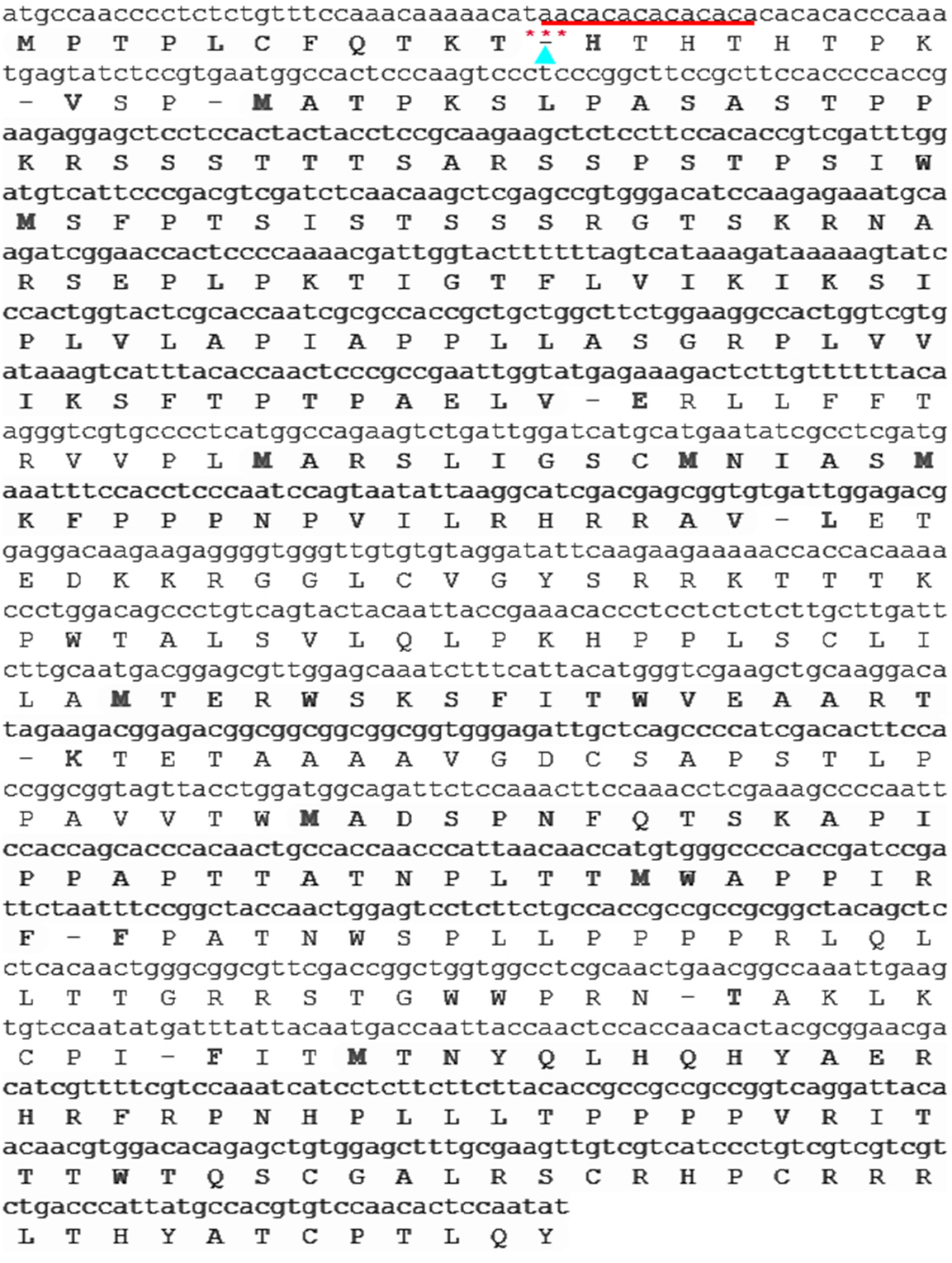

Supplement: supp_data_uhac136 [file supp_data_uhac136.zip › Fig. S4.tif]

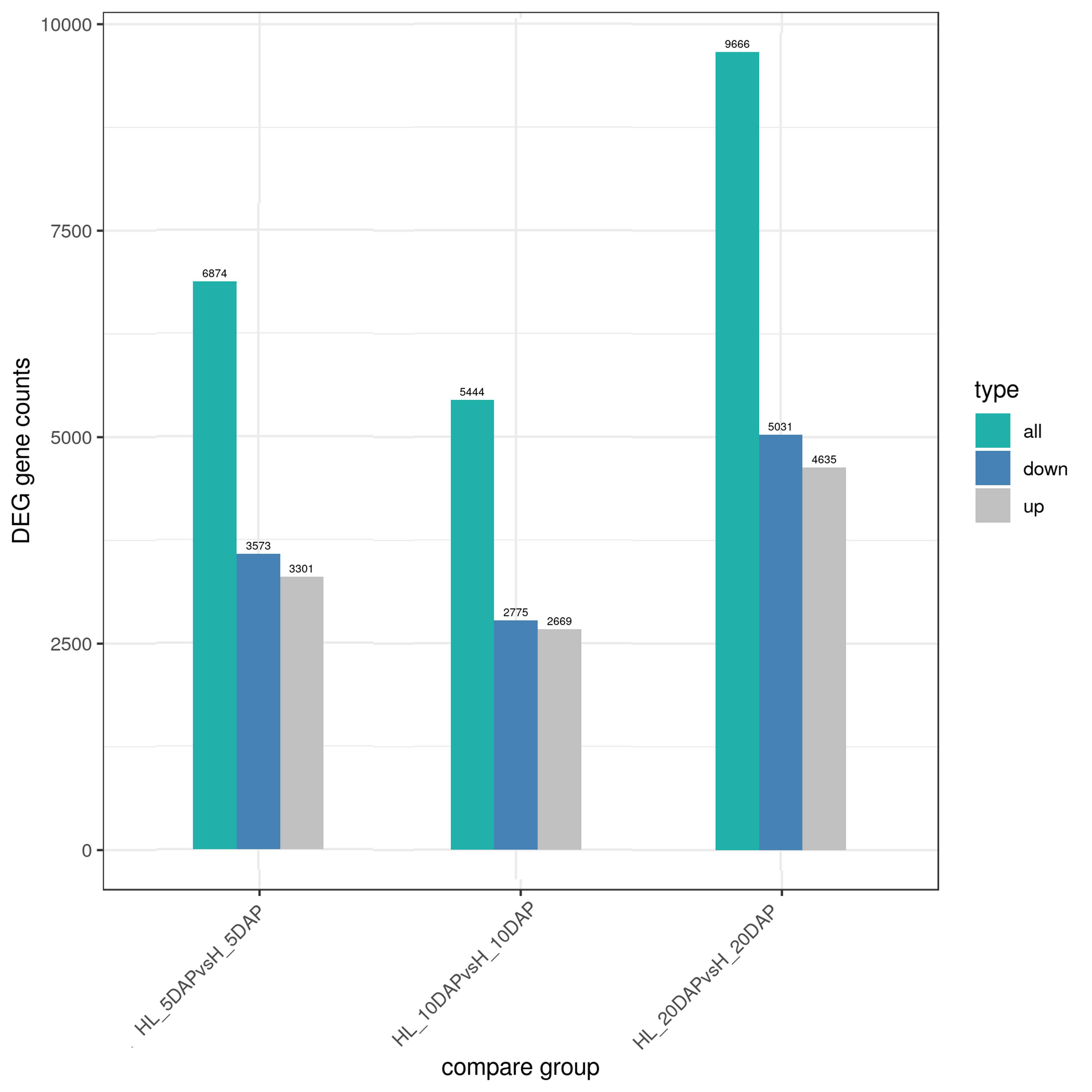

Supplement: supp_data_uhac136 [file supp_data_uhac136.zip › Fig. S5.tif]
